# Effect of Levodopa‐carbidopa Intestinal Gel on Non‐motor Symptoms in Patients with Advanced Parkinson's Disease

**DOI:** 10.1002/mdc3.12526

**Published:** 2017-09-20

**Authors:** David G. Standaert, Ramon L. Rodriguez, John T. Slevin, Michael Lobatz, Susan Eaton, Krai Chatamra, Maurizio F. Facheris, Coleen Hall, Kavita Sail, Yash J. Jalundhwala, Janet Benesh

**Affiliations:** ^1^ University of Alabama at Birmingham Birmingham Alabama USA; ^2^ University of Central Florida Orlando FL USA; ^3^ University of Kentucky Medical Center Lexington KY USA; ^4^ Neurology Center of Southern California CA USA; ^5^ AbbVie Inc. North Chicago IL USA

**Keywords:** LCIG, Parkinson's disease, Levodopa, non‐motor symptoms, quality of life

## Abstract

**Background:**

Levodopa‐carbidopa intestinal gel (LCIG; carbidopa‐levodopa enteral suspension in the United States), delivered via percutaneous gastrojejunostomy (PEG‐J) and titrated in the inpatient setting, is an established treatment option for advanced Parkinson's disease (PD) patients with motor fluctuations. However, long‐term prospective data on the efficacy of LCIG on non‐motor symptoms and the safety of outpatient titration are limited.

**Methods:**

In this 60‐week, open‐label phase 3b study, LCIG titration was initiated in an outpatient setting following PEG‐J placement in PD patients. The efficacy of LCIG on motor and non‐motor symptoms, quality of life, and safety was assessed.

**Results:**

Thirty‐nine patients were enrolled in the study and 28 patients completed the treatment. A majority of patients (54%) completed outpatient titration within the first week of LCIG infusion. LCIG led to significant reductions from baseline in Non‐Motor Symptom Scale (NMSS) total score (least squares mean ± SE = −17.6 ± 3.6, *P* < 0.001) and 6 of the NMSS domain scores (sleep/fatigue, attention/memory, gastrointestinal tract, urinary, sexual function, miscellaneous) at week 12. These reductions were maintained at week 60 with the exception of the urinary domain. “Off” time (−4.9 ± 0.5 hours/day, *P* < 0.001) and “On” time without troublesome dyskinesia (−4.3 ± 0.6 hours/day, *P* < 0.001) were improved at week 60. Adverse events (AEs) were reported in 37 (95%) patients.

**Conclusions:**

LCIG treatment led to reductions in non‐motor symptom burden and motor fluctuations in advanced PD patients. The safety profile was consistent with previous studies that used inpatient titration and outpatient titration did not appear to pose additional risk.

Levodopa‐carbidopa is the most efficacious drug treatment for Parkinson's disease (PD). However, disease progression and prolonged use of standard oral formulations are associated with the development of disabling motor complications that are refractory to medication changes in a majority of levodopa‐carbidopa‐treated PD patients. Fluctuations in response are driven primarily by non‐physiologic pulsatile stimulation of striatal dopamine receptors, which in turn are related partly to the progressive degeneration of dopaminergic neurons, the short half‐life of levodopa, and increasingly irregular gastric emptying.^1^, ^2^, ^3^, ^4^, ^5^, ^6^, ^7^ These fluctuations are also associated with a variety of non‐motor symptoms that substantially impact quality of life.^8^, ^9^, ^10^

Levodopa‐carbidopa intestinal gel (LCIG; carbidopa‐levodopa enteral suspension in the United States) provides continuous intestinal levodopa infusion via percutaneous endoscopic gastrojejunostomy (PEG‐J), and reduces fluctuations in levodopa plasma concentrations.^11^, ^12^ In prior phase 3 studies, including a 12‐week, double‐blind, double‐dummy clinical trial,^13^ LCIG reduced motor complications commonly associated with prolonged oral levodopa therapy in PD patients.^13^, ^14^, ^15^ Previously published data from observational studies also indicate that LCIG treatment is effective for the management of some non‐motor symptoms in PD patients refractory to oral levodopa therapy.^16^, ^17^ This open‐label, baseline‐controlled, phase 3b study evaluated the long‐term efficacy of LCIG on non‐motor symptoms in advanced PD patients over 60 week follow‐up.

In the previous phase 3 studies participants were hospitalized for up to 14 days for PEG‐J placement and LCIG dose titration; however, current clinical practice does not require routine hospitalization for these procedures. In this open‐label study, PEG‐J placement was performed as per clinical practice and LCIG dose titration was completed as an outpatient procedure.

## Methods

The efficacy of LCIG for the treatment of non‐motor symptoms and safety of outpatient titration were assessed in advanced PD patients experiencing motor fluctuations despite optimized medical therapy in this open‐label, phase 3b, 60‐week study (http://www.clinicaltrials.gov: NCT01736176). The study was conducted at 12 specialized movement disorder centers in the United States. The study protocol was approved by the institutional review board/ethics committee at each study center and all patients provided written informed consent.

### Patients

Eligible participants were advanced PD patients, ≥30 years old, levodopa‐responsive, and diagnosed with idiopathic PD. Eligible participants also had motor fluctuations with ≥3 hours of “Off” time per day at baseline despite individually optimized PD therapy and had recognizable “Off” and “On” mobility states, as observed by the study investigator and confirmed by PD diary records. Key exclusion criteria included an unclear PD diagnosis, a history of neurosurgical PD treatment, a Mini‐Mental State Examination score <24, or other evidence of significant cognitive impairment.

### Study Design

**Figure 1:**
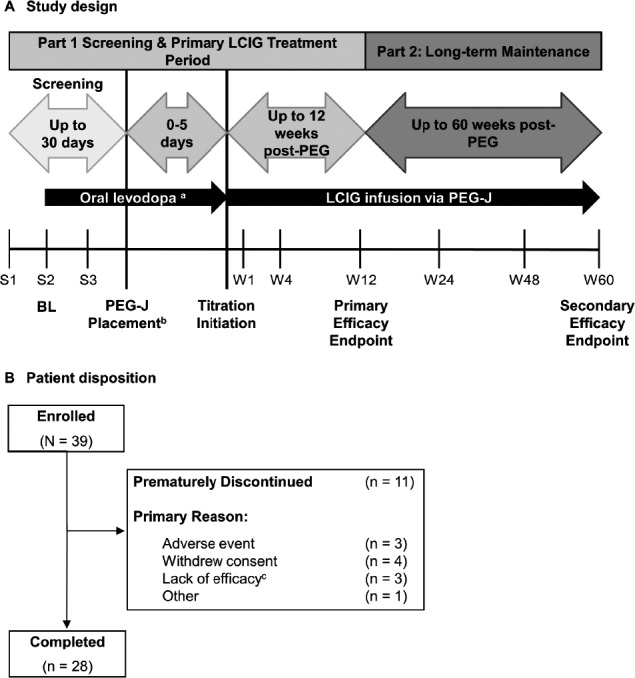
(**A**) Study design and (**B**) Patient disposition. Abbreviations: S, screening visit; W, week. ^a^Levodopa‐carbidopa immediate release tablets; ^b^Procedure was performed as a standard outpatient procedure by gastrointestinal specialists; if the investigator or gastrointestinal specialist considered it medically necessary, patients were observed for up to 48 hours post‐PEG‐J placement in an inpatient setting; ^c^2 patients who had lack of efficacy as their primary reason for discontinuation had an adverse event as additional reason for discontinuation.

The study included a screening period (≤30 days), PEG‐J placement, outpatient LCIG titration, a 12‐week primary LCIG treatment period, and a 48‐week, long‐term LCIG‐treatment maintenance period (Fig. 1A). Following baseline evaluation, all patients were converted from their carbidopa/levodopa doses (immediate or extended release) to oral study drug (carbidopa/levodopa 25/100 mg immediate release). All other anti‐PD medications were tapered‐off/discontinued at the time of LCIG initiation. Outside of the 16‐waking hours of continuous LCIG infusion, patients had the option to take oral carbidopa/levodopa. Following the 12‐week primary LCIG‐treatment period, additional concomitant anti‐PD medications could be introduced, if clinically indicated.

All patients received a 15 French Freka PEG tube and a 9 French Freka jejunal extension tube (Fresenius Kabi). The PEG‐J tube placement procedure occurred on study day 1 (D1) by a gastroenterologist, surgeon, or interventional radiologist as an outpatient procedure. At the discretion of the investigator or the physician, patients could require in‐patient (24–48 hours) observation.

LCIG initiation and outpatient dose titration began within 5 days of PEG‐J placement; outpatient summaries were based on data observed by clinical nurse educators rather than study investigators. LCIG was administered during waking hours; a morning dose/bolus was followed by continuous infusion for approximately 16 hours. The LCIG morning dose volume, continuous infusion rate, and rescue dose volume were programmed on the infusion pump at clinic visits. Pump settings were individually titrated based on clinical effect throughout the study. The LCIG dose was considered optimized when the programmed pump settings remained unchanged for 7 consecutive days. Patients controlled the daily start and stop times of infusions and could self‐administer additional rescue doses, if clinically indicated. Patients who completed their week 60 study visit before LCIG was commercially available to them had the option to remain in the study with clinic visits every 12 weeks.

### Efficacy

The primary efficacy outcome was the mean change in Non‐Motor Symptom Scale (NMSS) total score from baseline to week 12. The NMSS assesses non‐motor symptoms in PD, is obtained through patient interview, and contains 30 questions (grouped into 9 domains) that are scored with respect to severity. Total non‐motor symptom burden is defined as mild (NMSS total score = 1–15), moderate (16–40), severe (41–65), and very severe (≥66).^18^ Secondary efficacy analyses included the mean change from baseline at additional time points in NMSS total score, the 9 NMSS domain scores, PD Symptom Diary measures, the Unified Parkinson's Disease Rating Scale (UPDRS), the CANTAB spatial working memory (SWM) assessment, and the Controlled Oral Word Association Test (COWAT). The CANTAB and COWAT assessments were not required to be performed. Health related quality of life (HRQoL) outcomes were measured by the Parkinson's Disease Questionnaire‐39 Item (PDQ‐39) Summary Index (SI; score range = 0–100, higher scores indicate worse HRQoL), and the Patient Global Impression of Change (PGIC). For the PD Symptom Diary, patients recorded their motor states every 30 minutes throughout the waking day over the 3 consecutive days preceding each scheduled visit. Efficacy assessments were collected at baseline (screening visit 2, before conversion to oral levodopa monotherapy) and at preset intervals through 60 weeks.

### Safety

Treatment‐emergent adverse events (AEs) were documented throughout the study and included all AEs with onset on or after the date of PEG placement and within 30 days of the end of LCIG treatment. AEs were coded using the Medical Dictionary for Regulatory Activities 18.1 (MedDRA)^19^ and were tabulated by MedDRA preferred term (PT). Each safety event was coded to only one PT. For each AE, the study investigator rated it as ‘serious’ or ‘non‐serious’ and determined its relationship to LCIG treatment. For the assessment of treatment relationship, LCIG was considered as a therapeutic system consisting of drug, devices, and placement procedures. Causality assessments were to be made over the system as a whole. AEs of special interest included gastro‐intestinal (GI) and GI procedure‐related events, respiratory tract aspiration events, weight loss events, cardiac fatalities, and polyneuropathy events. AEs related to polyneuropathy were assessed by a MedDRA PT search strategy defined by the polyneuropathy and Guillain‐Barre syndrome Standardized MedDRA Queries (SMQs). A standardized panel of examination was suggested to the investigator in the event that a patient developed signs and symptoms of polyneuropathy; patients with polyneuropathy were treated at the investigators' discretion. To evaluate the effects of outpatient titration, AEs with onset during the first 4 weeks were summarized separately. Clinical laboratory, ECG, and vital signs were collected throughout the study.

### Statistical Analysis

Efficacy analyses included all patients who received at least 1 LCIG infusion and had baseline and post‐baseline observations for at least one efficacy or health outcome measure (N = 38). Safety summaries included all patients who underwent PEG‐J procedure (N = 39). Baseline was defined as the last non‐missing observation that was on or before the date of the patient's first oral study drug dose (efficacy) or before the date/time of the PEG‐J procedure (safety). Final visit was defined as the last non‐missing observation that was ≤1 day after last LCIG infusion (efficacy) or end of PEG‐J exposure (safety). For efficacy analyses, change from baseline was evaluated with a repeated measures model that included fixed effects of study site and visit, with baseline score as a covariate, and the baseline‐by‐visit interaction (reported as the least‐squares mean change ± standard error). PD Diary data were normalized to a 16‐hour waking day and averaged for the 3 days prior to each visit. Only diaries with ≥12 hours of awake‐time were included in the analysis. “On” time without troublesome dyskinesia (TSD) was defined as the sum of “On” time without dyskinesia and “On” time with non‐TSD.

## Results

Of the 39 patients enrolled, 38 had successful PEG‐J placement and received LCIG infusion and 28 (72%) completed the study (Fig. 1B). Eleven (28%) patients prematurely discontinued the study. At baseline, patients had a mean ± SD age of 64.3 ± 10.2 years and the mean PD duration was 11.5 ± 5.3 years. Patient demographics, PD characteristics, and baseline assessments are summarized in Table 1.

**Table 1:** Baseline demographics and disease characteristics

Characteristic	Value (N = 38)
Age, years, mean ± SD [range]	64.3 **±** 10.2 [43‐84]
Sex, female, n (%)	16 (41)
Race, white, n (%)	36 (92)
PD duration, years, mean ± SD	11.5 ± 5.3
NMSS total score,^a^ mean ± SD	48.3 ± 35.6
NMSS domains,^a^ mean ± SD	
Cardiovascular (including falls)	1.4 ± 2.1
Sleep/Fatigue	11.6 ± 9.2
Mood/Cognition	4.1 ± 6.2
Perceptual Problems/Hallucinations	1.9 ± 3.8
Attention/Memory	4.6 ± 6.4
Gastrointestinal Tract	5.3 ± 6.1
Urinary	8.3 ± 8.4
Sexual Function	2.7 ± 3.6
Miscellaneous^b^	8.3 ± 9.4
“Off” time,^a^ hours per day, mean ± SD	6.6 ± 1.6
“On” time with TSD,^a^ hours per day, mean ± SD	0.9 ± 1.8
“On” time without TSD,^a^ hours per day, mean ± SD	8.4 ± 2.2
PDQ‐39 SI, mean ± SD	34.7 ± 13.0
PD medications reported at baseline in > 5 patients, n (%)	
Levodopa	38 (100)
Amantadine	12 (32)
Pramipexole	10 (26)
Entacapone	8 (21)
Rotigotine	8 (21)
Ropinirole	6 (16)

NMSS, non‐motor symptom scale; PD, Parkinson's disease; PDQ‐39 SI, Parkinson's Disease Questionnaire 39‐item Summary Index; SD, standard deviation; TSD, troublesome dyskinesia

a n = 38

b Includes pain, taste/smell, weight, and excessive sweating

Following PEG‐J placement procedure, the mean ± SD time to patient discharge from the hospital/clinic was 20.4 ± 14.6 hours (N = 30); 63% (n/N = 19/30) of patients were discharged from the hospital within 24 hours. During outpatient titration, 66% (n = 25) of patients received their first LCIG dose within 24 hours of PEG‐J placement; 90% (n = 34) of patients had LCIG initiated within 48 hours. A majority of patients (n = 20/37, 54%) reached an optimized dose within the first week of LCIG infusion and only 2 (5.4%) patients required more than 2 weeks to achieve dose optimization. One patient discontinued treatment due to a non‐procedure/device‐related AE with onset during the first 4 weeks of LCIG infusion and 35 (92%) patients were still receiving LCIG infusions at the 12‐week primary endpoint. The mean ± SD time of patient exposure to LCIG was 427.2 ± 188.7 days (range 2–757 days; N = 38). The mean ± SD time of patient exposure to the LCIG delivery device was 427.5 ± 190.8 days (range 1–773 days; N = 39). Sixteen (42%) patients received concomitant anti‐PD medication during the 60‐week treatment period and 7 (18%) of these patients received non‐levodopa medication. The most common concomitant, non‐levodopa, anti‐PD medication during the 60‐week treatment phase was entacapone (n = 4, 10.5%).

### Efficacy

**Figure 2:**
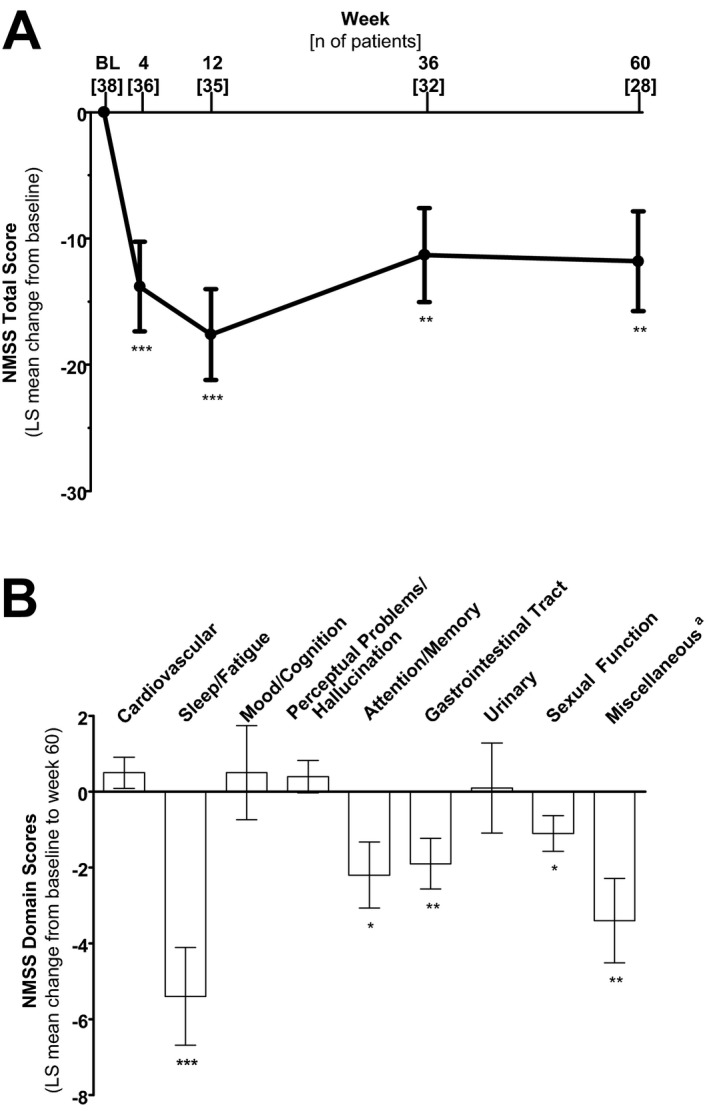
(**A**) Least squares mean change from baseline in the NMSS total score and (**B**) Least squares mean change in the NMSS domain scores at week 60. At week 60, n = 28. Error bars indicate standard error. *P*‐values from a repeated measure model that included fixed effects of study site and visit, with baseline score as a covariate, and the baseline‐by‐visit interaction indicate statistically significant mean change from baseline scores of *P* < 0.05 (*), *P* < 0.01 (**) and *P* < 0.001 (***). Abbreviations: LS, least squares; NMSS, Non‐Motor Symptom Scale. ^a^Includes pain, taste/smell, weight, and excessive sweating.

On average, patients had severe non‐motor symptom burden at baseline (NMSS total score, mean ± SD = 48.3 ± 35.6).^18^ At the primary 12‐week endpoint, LCIG‐treated patients showed significant reductions in NMSS total score with a least squares (LS) mean ± SE change of −17.6 ± 3.6 (*P* < 0.001) (Fig. 2A). NMSS total score remained significantly reduced at every study visit from baseline through week 60 (Table 2). For patients with severe non‐motor symptom burden at baseline (baseline NMSS total score > 40), the primary efficacy outcome was similar to the overall patient population (NMSS total score, LS mean change from baseline ± SE: week 12 [n/N = 16/35] −33.4 ± 6.2; week 60 [n/N = 12/28], −25.7 ± 6.9). Five of the 9 NMSS domain scores were significantly decreased compared to baseline at week 60 (sleep/fatigue, *P* < 0.001; attention/memory, *P* = 0.013; gastrointestinal tract, *P* = 0.006; sexual function, *P* = 0.021; miscellaneous, *P* = 0.003). The NMSS urinary domain was significantly reduced compared to baseline at week 12 (*P* = 0.044; Fig. 2B; Table 2). LCIG treatment also led to significant improvements in the PDQ‐39 SI at every study visit through week 60 (Table 3). Additionally, a majority of patients considered their status to be improved (minimally improved, much improved, or very much improved) at week 12 (PGIC, n/N = 30/38, 80%) and week 60 (PGIC, n/N = 27/38, 71%); no patients considered their status as much worse or very much worse at these time points. Changes in neurocognition, measured using the CANTAB SWM and COWAT, were not observed at week 12 or week 60 (week 60: CANTAB SWM, mean change from baseline ± SD = −0.6 ± 7.7, *P* = 0.741, n = 18; COWAT, 0.8 ± 2.3, *P* = 0.144, n = 18).

**Table 2:** Least squares mean change from baseline on the Non‐Motor Symptom Scale total score and domains

	BL (n = 38)	Week 12 (n = 35)		Week 60 (n = 28)	
	Mean ± SD	Change from BL LS Mean ± SE	*P* Value	Change from BL LS Mean ± SE	*P* Value
NMSS total score^a^	48.3 ± 35.6	−17.6 ± 3.6	<0.001	−11.8 (4.0)	0.004
NMSS domains^a^					
Cardiovascular^b^	1.4 ± 2.1	−0.2 ± 0.4	ns	0.5 ± 0.4	ns
Sleep/Fatigue	11.6 ± 9.2	−6.0 ± 1.2	<0.001	−5.4 ± 1.3	<0.001
Mood/Cognition	4.1 ± 6.2	0.0 ± 1.1	ns	0.5 ± 1.2	ns
Perceptual problems/Hallucinations	1.9 ± 3.8	−0.5 ± 0.4	ns	0.4 ± 0.4	ns
Attention/Memory	4.6 ± 6.4	−2.1 ± 0.8	0.010	−2.2 ± 0.9	0.013
Gastrointestinal tract	5.3 ± 6.1	−2.0 ± 0.6	0.001	−1.9 ± 0.7	0.006
Urinary	8.3 ± 8.4	−2.2 ± 1.1	0.044	0.1 ± 1.2	ns
Sexual function	2.7 ± 3.6	−1.8 ± 0.4	<0.001	−1.1 ± 0.5	0.021
Miscellaneous^c^	8.3 ± 9.4	−3.4 ± 1.0	0.001	−3.4 ± 1.1	0.003

BL, baseline; LS, least squares; NMSS, non‐motor symptom scale; ns, not significant; SD, standard deviation; SE, standard error.

a The NMSS contains 30 questions grouped into 9 domains. Each question is scored with respect to severity (range from 0 = none to 3 = severe) and frequency (range from 1 = rarely to 4 = very frequent). Item scores are calculated as the product of severity and frequency. A negative NMSS score indicates improved symptom severity. NMSS total score and NMSS domains were analyzed by a mixed‐effect model for repeated measures using factors of study site, visit, and baseline, and the baseline‐by‐visit interaction.

b Including falls

c The Miscellaneous domain includes questions on pain, change in the ability to taste and/or smell, weight change and excessive sweating.

**Table 3:** Least squares mean change from baseline in the UPDRS total score, UPDRS part II, III, and IV scores, and the PDQ‐39 Summary Index^a^

	Baseline N = 37	D7 N = 36	Week 12 N = 34	Week 36 N = 32	Week 60 N = 28
UPDRS total score^b^	43.3 ± 17.8	−8.8 ± 1.5***	−11.4 ± 1.7*	−7.1 ± 1.9***	−7.7 ± 2.3**
UPDRS I	1.6 ± 1.6	−0.6 ± 0.2**	−0.3 ± 0.3	−0.3 ± 0.3	−0.1 ± 0.3
UPDRS II	16.7 ± 6.5	−4.8 ± 0.7***	−5.5 ± 0.9***	−4.2 ± 0.9***	−4.7 ± 0.9***
UPDRS III	25.0 ± 13.2	−3.5 ± 1.2**	−5.6 ± 1.2***	−2.6 ± 1.5	−3.6 ± 1.5**
UPDRS IV^c^	8.7 ± 3.0	−2.7 ± 0.5***	−3.5 ± 0.4***	−3.5 ± 0.4***	−2.9 ± 0.6***
UPDRS dyskinesia items^d^	3.0 ± 2.1	−1.1 ± 0.4**	−1.1 ± 0.3***	−1.1 ± 0.3***	−0.6 ± 0.4
PDQ‐39 SI	34.7 ± 13.0	−4.8 ± 1.8*	−11.2 ± 2.8***	−9.1 ± 2.2***	−10.2 ± 2.6***

D, day; LS, least squares; PDQ‐39 SI, Parkinson's Disease Questionnaire 39‐tem Summary Index; UPDRS, Unified Parkinson's Disease Rating Scale; Values compared to baseline with paired t‐test, *P* < 0.001 (***), 0.01 (**), 0.05 (*).

a All values depict LS mean ± standard error with the exception of the baseline values which represent the mean ± standard deviation.

b UPDRS was collected in the “On” state.

c Baseline, n = 38; D7, n = 37; Week 12, n = 35.

d Items 32, 33, 34.

**Figure 3:**
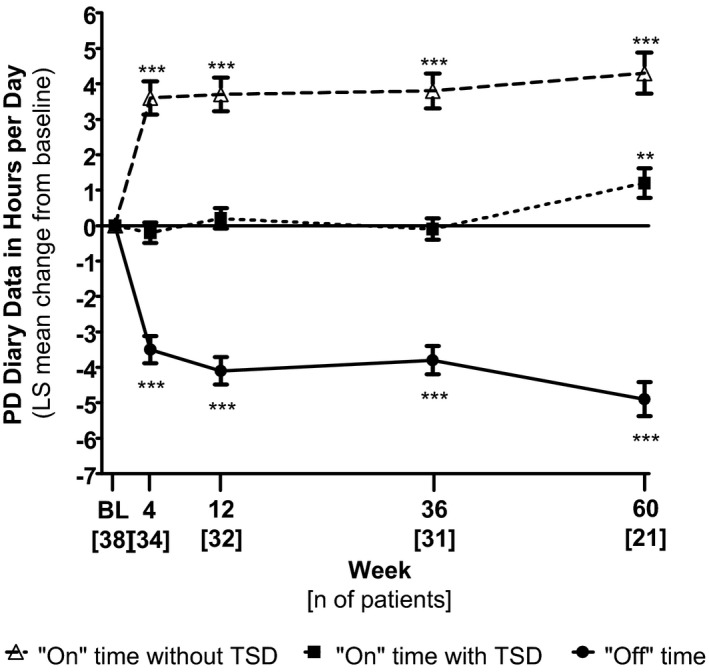
Least squares mean change from baseline in PD Symptom Diary measures over time. Error bars indicate standard error. *P*‐values from a repeated measure model that included fixed effects of study site and visit, with baseline score as a covariate, and the baseline‐by‐visit interaction indicate statistically significant mean change from baseline to final scores of *P* < 0.01 (**) and *P* < 0.001 (***). Abbreviations: BL, baseline; LS, least squares; PD, Parkinson's disease; TSD, troublesome dyskinesia

Significant and sustained reductions in mean hours of “Off” time were observed through week 60 (Fig. 3 and Table 4). “Off” time reductions were observed alongside increased “On” time without TSD. “On” time without TSD was significantly increased from baseline through week 60 (Fig. 3). “On” time with TSD was stable through week 36 and increased significantly at week 60 compared to baseline (*P* = 0.004). PD Diary asleep‐time was not significantly changed at week 60 (Table 4). LCIG‐treatment was also associated with significant and sustained improvement in the UPDRS total and parts II, III, and IV scores and UPDRS dyskinesia items 32, 33, and 34 (Table 3).

**Table 4:** Least squares mean change from baseline in the PD diary measures^a^

	Baseline N = 38	D7 N = 34	Week 12 N = 32	Week 36 N = 31	Week 60 N = 21
‘Off’ time	6.6 ± 1.6	−1.4 ± 0.4***	−4.1 ± 0.4***	−3.8 ± 0.4***	−4.9 ± 0.5***
‘On’ time without TSD	8.4 ± 2.2	1.0 ± 0.5*	−3.7 ± 0.5***	−3.8 ± 0.5***	−4.3 ± 0.6***
‘On time with TSD	0.9 ± 1.8	0.3 ± 0.3	0.2 ± 0.3	−0.1 ± 0.3	1.2 ± 0.4**
Absolute asleep time	7.5 ± 1.8	0.0 ± 0.2	0.2 ± 0.2	−0.1 ± 0.3	0.3 ± 0.3

D, day; LS, least squares; PD, Parkinson disease; TSD, troublesome dyskinesia; Values compared to baseline with paired t‐test, *P* < 0.001 (***), 0.01 (**), 0.05 (*).

a Normalized to 16‐hour waking day; All values depict least squares mean ± standard error with the exception of the baseline values which represent the mean ± standard deviation.

### Safety

Overall, 37 (95%) patients experienced any AE and 35 (90%) patients experienced an AE assessed by the investigator as possibly related to LCIG (Table 5). The most common AEs were procedural pain (patient incidence = 33%) and stoma site infection (28%). AEs were generally mild to moderate in severity, with 5 (13%) patients reporting a severe AE. Severe AEs considered by the investigator to have a reasonable possibility of being related to LCIG were: major depression and suicidal ideation (n = 1), pneumonia and orthostatic hypotension (n = 1), peritonitis (n = 1), and anxiety (n = 1). Eight (21%) patients experienced a serious AE (SAE; Table 5).

**Table 5:** Safety summary

	Number of Patients (% of N = 39)
Any adverse event (AE)	37 (95)
During Weeks 1‐4	28 (72)
Any GI event^a^	28 (72)
During Weeks 1‐4	22 (56)
Any non‐GI event	37 (95)
During Weeks 1‐4	21 (54)
Any serious AE (SAE)	8 (21)
During Weeks 1‐4	3 (8)
Any AE leading to a discontinuation	5 (13)
During Weeks 1‐4	1 (3)
Death	1 (3)
During Weeks 1‐4	0
AEs Occurring in ≥ 10% Patients	
Procedural pain	13 (33)
Stoma site infection	11 (28)
Stoma site pain	9 (23)
Anxiety	8 (21)
Stoma site erythema	8 (21)
Fall	7 (18)
Weight decreased	7 (18)
Urinary tract infection	6 (15)
Orthostatic hypotension	5 (13)
Excessive granulation tissue	4 (10)
Flatulence	4 (10)
Nausea	4 (10)
Stoma site irritation	4 (10)
Vitamin B6 deficiency	4 (10)
SAEs Occurring in Any Patient	
Acute respiratory failure	1 (3)
Anxiety	1 (3)^b^
Atrial fibrillation	1 (3)^b^
Aspiration pneumonia	1 (3)
Basal cell carcinoma	1 (3)
Congestive cardiac failure	1 (3)
Internal hernia	1 (3)^b^
Major depression	1 (3)^b^
Osteoarthritis	1 (3)
Peritonitis	1 (3)^b^
Radiculopathy	1 (3)
Respiratory distress	1 (3)^b^
Sedation	1 (3)
Suicidal ideation	1 (3)^b^

AE, adverse event; GI, gastrointestinal; SAE, serious adverse event.

a GI events includes all MedDRA preferred terms in the GI and GI procedure related events query^.^

b Investigator determined SAE had a reasonable possibility of being related to treatment

Polyneuropathy‐related AEs were reported in 3 (8%) patients, were mild in severity, and included loss of proprioception (n = 1), peripheral neuropathy alone (n = 1), and peripheral neuropathy with peripheral sensory neuropathy (n = 1). Prior to LCIG initiation, patients reporting polyneuropathy‐related AEs were receiving between 1700 and 2400 mg daily levodopa dose (oral carbidopa/levodopa 25/100 mg immediate release). The reports of peripheral neuropathy alone and peripheral neuropathy with peripheral sensory neuropathy were deemed by the investigators to have a possible relationship to study drug. The patient who experienced a loss of proprioception had a history of peripheral nerve disorder and vitamin B12 deficiency; the event was determined by the investigator to have no relationship to study drug or oral carbidopa/levodopa immediate release. No polyneuropathy‐related AEs led to premature discontinuation of LCIG. Other AEs of special interest included GI and GI procedure‐related events (n = 28, 72%), weight loss events (n = 8, 21%), and respiratory tract aspiration events (n = 7, 18%).

During weeks 1–4, 22 (56%) patients experienced a GI‐related AE and 21 (54%) patients experienced a non‐GI related AE (Table 5). AEs that occurred in a higher percentage of patients during weeks 1–4, than during the longer LCIG treatment period (weeks 5–60) included procedural pain (weeks 1–4: 33%; weeks 5–60: 5.1%), anxiety (weeks 1–4: 13%; weeks 5–60: 10%) flatulence (weeks 1–4: 10%; weeks 5–60: 0%) and stoma site irritation (weeks 1–4: 7.7%; weeks 5–60: 2.6%). During weeks 1–4, 2 patients experienced SAEs possibly related to LCIG (peritonitis [n = 1], internal hernia [n = 1]).

Among the 5 patients (13%) who discontinued study drug due to AE, 4 discontinued due to events considered by the investigator to be possibly related to LCIG. One patient with a baseline Mini Mental State Examination score of 24 discontinued study drug on day 72 due to cognitive disorder with onset on treatment day 3 that remained unresolved; 1 patient discontinued due to stoma site pain with onset on day 130 that resolved 8 days later; 1 patient discontinued due to a stoma site infection with onset on day 255 that resolved 79 days later; 1 patient discontinued due to the onset of pneumonia on day 255 that lasted for 15 days; another patient discontinued study drug on day 178 due to events of congestive cardiac failure, acute respiratory failure, and aspiration pneumonia following spinal surgery. These events were considered to be unrelated to LCIG by the study investigator and resulted in the patient's death on day 179.

## Discussion

This open‐label, phase 3b study evaluated the long‐term efficacy of LCIG for the treatment of non‐motor symptoms and the safety of outpatient titration in advanced PD patients. Continuous infusion of LCIG led to significant and clinically meaningful^20^ reductions from baseline in non‐motor symptom severity at the primary 12‐week endpoint and these reductions were maintained through week 60. Importantly, LCIG treatment reduced the mean non‐motor symptom burden from a severe level at baseline to a moderate level by week 4; this moderate non‐motor symptom burden was maintained through week 60.^18^ NMSS domains that showed persistent improvement over 60‐weeks included the sleep/fatigue, attention/memory, gastrointestinal tract, sexual function, and miscellaneous domains. Disturbances in sleep/fatigue, which have a substantial impact on quality of life,^17^ showed the greatest magnitude of improvement at week 60 despite no statistically significant change in absolute sleep time. While significant improvement in the urinary domain at week 12 did not persist through week 60, previous studies on LCIG have demonstrated long‐term treatment‐related improvements in this NMSS domain.^16^, ^17^, ^21^

In addition to the observed non‐motor symptom improvements, LCIG treatment also led to a significant and sustained improvement in HRQoL. The improvements in non‐motor symptoms and HRQoL are consistent with previously published long‐term data on LCIG, including 12‐month data from the observational GLORIA study, which reported non‐motor symptom and HRQoL improvements in PD patients treated with LCIG during routine care.^16^, ^17^ Additionally, this study's results are consistent with a prospective observational study that reported improvement in non‐motor symptom severity with LCIG treatment that was superior to the effects observed with apomorphine.^21^

Reduced non‐motor symptom burden occurred alongside sustained improvements in motor symptoms, including a significant and clinically meaningful decrease in “Off” time through week 60.^22^ Notably, reduced “Off” time was observed together with a significant increase in “On” time without TSD. These data indicate that reduction in “Off” time was not achieved at the expense of increasing dyskinesia, an important consideration in efforts to improve patients' quality of life.^23^

A secondary objective of this study was to evaluate the safety of outpatient LCIG titration, an approach that is more reflective of current clinical practice. The data indicate the success of outpatient titration, with 90% of patients beginning LCIG infusion within the first 48 hours after PEG‐J placement and a majority of patients reaching LCIG dose optimization within one week of their first LCIG infusion. The safety results also suggest that outpatient LCIG titration was generally well tolerated. The percentage of patients in the current study that experienced an AE during the first 4‐weeks following PEG‐J placement (71.8%) was lower than previously reported in other phase 3 LCIG studies that utilized inpatient titration, including a 12‐week, double‐blind, placebo‐controlled, double‐dummy clinical trial (% of patients with AE during weeks 1–4 = 86.5%) and a 12‐month open label study (% of patients with AE during weeks 1–4 = 78.4%).^24^ A similar pattern was observed for SAEs, with a substantially lower percentage of patients reporting a SAE (7.7%) during weeks 1–4 post‐PEG‐J placement in the current study compared to previous phase 3 studies using inpatient titration (% of patients with SAE during weeks 1–4: double‐blind study = 13.5%, open‐label study = 13.5%).^24^ Additionally, the discontinuation rate during the first 4‐weeks following PEG‐J placement (2.6%) was comparable to the week 1–4 discontinuation rate reported in an integrated analysis of previous phase 3 studies (2.0%).^25^ In the current study, only one patient discontinued treatment due to an event with onset during the first 4‐weeks of LCIG infusion and the reason for discontinuation was unrelated to the procedure/device.

The safety results over the course of the entire study also speak to the general success of outpatient titration. While the discontinuation rate (28%) and rate of discontinuations due to AE (13%) over the entire study course were higher than previous phase 3 studies (overall phase 3 discontinuation rate = 17%,^25^ discontinuation due to AE rate from Fernandez et al. [2015] = 8%^15^), study discontinuations were distributed over the entire course of the study and likely not influenced by outpatient titration, which was completed within the first 4‐weeks of treatment. The percentage of patients that experienced ≥1 AE (95%) and the severity of these AEs (mostly mild/moderate) were comparable to previous reports.^15^, ^25^ Compared to previous phase 3 studies, the percentage of patients who experienced ≥ 1 GI‐related AE was slightly lower in the current study (56% vs. 62%).^15^ Polyneuropathy was identified in 3 (8%) patients, was mild in severity, non‐serious, and did not lead to premature discontinuation for any patient. Polyneuropathy‐related events occurred at a rate that is consistent with reports in the literature for PD patients receiving levodopa therapy as well as an integrated analysis of safety from previous phase 3 studies on LCIG.^26^ Also important to note is that one patient reporting a polyneuropathy‐related AE had a previous history of peripheral nerve disorder. Serious AEs (21%) did not indicate any new safety concerns for LCIG.

Limitations of this study include its open‐label design and lack of a control group, which is important to consider, as treatment‐related improvements in non‐motor symptoms have been associated with a strong placebo effect in recent literature.^27^ However, the long‐term follow‐up, magnitude of observed change in non‐motor symptom burden, and the similarity of the current findings to previous data on LCIG efficacy for the treatment of non‐motor symptoms in advanced PD patients, are strengths of the current study.

In summary, LCIG demonstrated sustained and clinically meaningful reductions in non‐motor symptom burden and improvements in quality of life in advanced PD patients with motor fluctuations over a 60‐week treatment period. Also, LCIG was generally well tolerated, supporting the established profile of LCIG and the safety of outpatient titration.

## Author Roles

1. Study Concept/design; 2. Data Acquisition; 3. Statistical Analysis; 4. Data Interpretation.

D.G.S: 1, 2, 4

R.L.R.: 2, 4

J.T.S.: 2, 4

M.L.: 2, 4

S.E.: 1, 2, 4

K.C.: 1, 2, 4

M.F.: 2, 4

C.H.: 1, 2, 3, 4

K.S.: 4

Y.J.J.: 4

J.B.: 1, 2, 4

## Disclosures

**Ethical Compliance Statement**: We, the authors, confirm that we have read the Journal's position on issues involved in ethical publication and affirm that this work is consistent with those guidelines.

**Funding Sources and Conflict of Interest**: Study‐related disclosures: D.G.S., R.L.R., J.T.S., and M.L. were study investigators. D.G.S., J.T.S. were paid consultants to AbbVie for study design and medical/scientific advice. J.T.S. received research support from AbbVie. S.E. and K.C. are former AbbVie employees. M.F., C.H., K.S., Y.J.J., and J.B. are employees of AbbVie and own AbbVie stock/stock options.

This work was funded by AbbVie Inc. AbbVie participated in the study design, research, data collection, analysis, and interpretation of data, writing, reviewing, and approving the publication. Medical writing support was provided by Amy M. Spiegel of AbbVie Inc.

**Financial disclosures from previous 12 months**:** D.G.S.** is a member of the faculty of the University of Alabama at Birmingham and is supported by endowment and University funds. Dr. Standaert is an investigator in studies funded by Abbvie, Inc., the American Parkinson's Disease Association, The Michael J. Fox Foundation for Parkinson's Research, Alabama Department of Commerce, and NIH grants P01NS087997, P20NS087997, R25NS079188, P2CHD086851, and P30NS047466. He has a clinical practice and is compensated for these activities through the University of Alabama Health Services Foundation. In addition, since January 1, 2016 he has served as a consultant for or received honoraria from, Serina Therapeutics, Abbvie, Voyager Therapeutics, the Michael J. Fox Foundation for Parkinson's Research, The International Parkinson's Disease and Movement Disorder Society, the National Institutes of Health, The American Institute for Biological Sciences, Rush University, Huntsville Hospital, UCSD, Voyager Therapeutics, and he has received royalties for publications from McGraw Hill, Inc. R.L.R. has received research support from AbbVie Inc., Allergan, Auspex, Biotie Therapeutics, Dystonia Coalition, Huntington Study Group, Ipsen, Merz Pharmaceuticals, National Parkinson's Foundation, NIH/NINDS, Parkinson's Study Group, Chelsea, and Auspex, but has no owner interest in any pharmaceutical company; honoraria from PeerView Institute for Medical Education, Merz, Lundbeck, Chelsea, and the CME Meeting; and honoraria from AbbVie Inc. for speaking and educational symposium. J.T.S. has served as an advisor for AbbVie Inc. and Cynapsus Therapeutics Inc., a speaker for Teva and AbbVie Inc., and has received research support from AbbVie Inc., Biotie, NIH, Parkinson's Study Group and Veterans Administration. M.L. was a study investigator. M.F.F., C.H., K.S., Y.J.J., and J.B. are employees of AbbVie Inc and hold AbbVie Inc. stock and/or stock options. K.C. and S.E. are former employees of AbbVie Inc.
